# Partial Androgen Deficiency, Depression, and Testosterone Supplementation in Aging Men

**DOI:** 10.1155/2012/280724

**Published:** 2012-06-07

**Authors:** Mario Amore, Marco Innamorati, Sara Costi, Leo Sher, Paolo Girardi, Maurizio Pompili

**Affiliations:** ^1^Department of Neurosciences, Institute of Psychiatry, University of Parma, Piazza Matteotti 9, 43100 Parma, Italy; ^2^Department of Psychiatry, Mount Sinai School of Medicine, New York, NY 10029, USA; ^3^Department of Neurosciences, Mental Health and Sensory Organs, Suicide Prevention Center, Sant'Andrea Hospital, Sapienza University of Rome, 00189 Rome, Italy

## Abstract

The aim of this review was to summarize current knowledge on the correlation between depressive symptoms with a syndrome called partial androgen deficiency of the aging male (PADAM) and on the potential benefits of testosterone (T) treatment on mood. Despite, the causative nature of the relationship between low T levels and depression is uncertain, many hypogonadal men suffer from depression and vice versa several depressed patients are affected by hypogonadism. Supplementation with testosterone failed to show sound evidence of effectiveness in the treatment of depression. Nevertheless, testosterone supplementation has proved to be effective on some domains significant for the quality of life of aged patients with PADAM (sexual function and cognitive functions, muscular strengths).

## 1. Introduction

Testosterone deficiency or hypotestosteronemia is a widely recognized hormonal alteration associated with male aging [1–3]. Its prevalence may be as high as 30% in men aged 40–79 years [4, 5], and in up to 12% the hypotestosteronemia it can be associated with clinical symptoms [5]. Nevertheless, different levels of testosterone (T) could be associated with the presence of specific clinical symptoms [6, 7].

The joint consensus of International Society of Andrology, the International Society for the Study of the Aging Male (ISSAM) and the European Association of Urology prepared a set of recommendations specifically on the Investigation, treatment, and monitoring of late-onset hypogonadism in males [8, 9]. Laboratory diagnosis of hypogonadism is based on the measurement of serum total testosterone (TT). Although there is no uniformly accepted threshold level for T in older men, TT levels above 350 ng/dL are considered normal and do not require substitution therapy, while TT levels below 230 ng/dL usually benefit from testosterone treatment. When TT level is between 230 and 350 ng/dL, it may be useful to calculate free testosterone (FT), particularly in obese men. FT level below 65 pg/mL suggests that testosterone treatment is needed [10, 11].

In 2010, the Endocrine Society published clinical practice guidelines for testosterone therapy in adult men with androgen deficiency syndrome [12]. The members of the working group agreed that because the normative ranges for TT and FT in healthy young men vary among laboratories and assays (lower TT limits: 280–300 ng/dL; lower FT limits: 5–9 pg/mL) [13], clinicians should use the lower limit of normal range for healthy young men established in their laboratory. Members of the working group disagreed on T concentrations below which testosterone supplementation should be offered to older men with symptomatic hypogonadism. Some members of the working group recommended T supplementation in older men with TT level below 300 ng/dL, because this is the threshold at which older men have symptoms that might be attributable to low testosterone; others recommended T supplementation only in those with TT level below 200 ng/dL, because higher pretreatment T values are associated with lower beneficial effects of T therapy.

Age-related serum testosterone decline is caused by different simultaneous mechanisms, such as primary structural gonadal impairment, age-related degenerative modifications of the pituitary gland, deficits of the neurohypothalamic system, and primary peripheral metabolic abnormalities such as the age-associated increase in the concentration of serum sex hormone binding globulin (SHBG), with a consequent decrease in FT [3].

It is controversial whether aging is to be considered as the only variable linked to age-related T decline [14, 15]: several factors do seem to interfere in different ways with T metabolism, like genetic factors [16], chronic diseases [17–19], chronic medications [20], obesity [7, 21, 22], and lifestyle factors [23, 24].

Despite the fact that many men with low testosterone levels are asymptomatic [25], many others have a partial, gradual, and variable decline in T associated with various clinical symptoms, described as a syndrome called partial androgen deficiency of the aging male (PADAM) [26]. PADAM is characterized by sexual, somatic, and behavioral symptoms, with insidious onset and slow progression [27]: diminished sexual desire and erectile quality, particularly nocturnal erections [28, 29]; decrease in lean body mass, with associated diminution in muscle volume and strength; increase in visceral fat [30–32], decrease in bone mineral density, resulting in osteoporosis [33]; reduction in body hair and skin alterations [34]; weakness, fatigue, depression, lack of motivation and energy, lower psychological vitality, anxiety, irritability, insomnia, decreased work and sport performances; difficulty in concentrating, memory impairment, and low dominance [35–41].

In the Endocrine Society Guidelines symptoms are separated into two groups, more specific symptoms and signs of hypogonadism (incomplete or delayed sexual development, sexual disorders, breast discomfort, gynecomastia, loss of body [axillary and pubic] hair, reduced shaving, very small or shrinking testes, inability to father children, low or zero sperm count, height loss, low trauma fracture, low bone mineral density, hot flushes, and sweats) and less specific symptoms (decreased energy, motivation, initiative, self-confidence, feeling sad or blue, depressed mood, dysthymia, poor concentration and memory, sleep disturbance, increased sleepiness, mild anemia, reduced muscle bulk and strength, increased body fat, body mass index, and diminished physical or work performance) [12]. Serum T concentration has to be measured in patients with the more specific symptoms of hypogonadism and considered in those who report the less specific symptoms. The diagnosis of hypogonadism is possible when serum T level is below lower limits, and reversible illness, drugs, and nutritional deficiency have been excluded.

PADAM as a clinical entity is still controversial, because it is very difficult to distinguish to what extent the symptoms attributed to PADAM are due to the natural and unavoidable consequences of aging and how much to androgen deficiency [37, 42, 43].

Behavioral aspects of PADAM may overlap with signs of depression. For example, McIntyre et al. [44] considered that reduction in physiologically active bioavailable testosterone (BT) concentration is a vulnerability factor for depressive symptoms in middle-aged depressed men. The authors assessed and compared TT and BT levels in two groups of middle-aged men (40–65 years), untreated subjects meeting DSM-IV-TR (Diagnostic and Statistical Manual of Mental Disorders—4th edition, text revised) [45] criteria for a major depressive episode (*N* = 44), and a matched nondepressed control group (*N* = 50). Depressed men had lower mean BT levels and TT levels than the control group. Biochemical hypogonadism (i.e., BT level ≤70 ng/dL or TT level ≤350 ng/dL) was also more prevalent in depressed men than in nondepressed controls (34% versus 6%; 61% versus 14%, resp.).

Thus, the aim of this review was to summarize current knowledge on depressive symptoms correlated with PADAM and on the potential benefits of T treatment on mood.

## 2. Methods

In order to provide a critical review of the association of PADAM and depression in older males, we performed a PubMed search to identify all papers published in English peer-reviewed journals between 1980 and 2012. The search string was androgen deficiency OR testosterone deficiency OR hypogonadism OR testosterone treatment OR testosterone supplementation AND depress*.

We limited the search to articles reporting data for male aged 45+ years old. All English full-text articles reporting original data about the main topic were included. The reference lists of the articles included in the review were also manually checked to retrieve other relevant studies.

## 3. Results

### 3.1. T Levels and Depression

Epidemiological and clinical studies of the connection between age-related low T levels and a reduced feeling of well-being, with unusual anxiety and irritability, nervousness, mood swings, and a depressive state, have produced mixed results [46–63] (see Tables 1 and 2).

Positive results were reported by Hintikka et al. [47] who examined associations between hypogonadism (laboratory diagnosis was based on FT level <4.6 ng/dL), erectile dysfunction, sexual desire, and long-term and current depressive symptoms in a population-based sample of Finnish middle-aged men. The inclusion criteria for this study were based on self-reported adverse mental symptoms prevailing at baseline and at the 3-year followup. At 7 years from the baseline, men who reported long-term adverse mental symptoms had higher depression but lower FT levels than asymptomatic men. Furthermore, depression correlated negatively with FT (rho = −0.20; *P* < 0.05) in the entire sample.

The Rancho Bernardo Study examined the association between T and depression in 856 community-dwelling older men aged 50 to 89 years (mean age 70.2 years) during a period of 4 years [64]. In that study, BT levels decreased with age and were significantly and inversely associated with Beck Depression Inventory (BDI) scores [65], indicating more depressive symptoms associated with lower BT levels. There was a graded stepwise decrease in BT, with a parallel increasing level of depressed mood; in addition, BT levels were 17% lower in men with categorically defined depression than in controls.

Lee et al. [66] investigated the PADAM in a community sample of 311 Chinese men (aged 40–80) attending a family medicine clinic in Hong Kong. A total of 87.8% of the sample was screened PADAM positive using the ADAM questionnaire. PADAM-positive individuals were found to have poorer quality of life, higher depression and anxiety, even after adjusting for age and number of current diseases.

In a historical cohort study, the Veterans Affairs Puget Sound Health Care System carried out on 278 men aged 45 years and older without previous depressive diagnosis, hypogonadal men with TT levels of 200 ng/dL or less (compared to eugonadal men) showed an approximate 4-fold increase in the risk of incident depression in the 2-year followup [67]. The risk of depression was inversely related to T levels, with statistically significant findings observed at T levels lower than 280 ng/dL.

In a sample of 32 subjects with dysthymic disorder (mean age 70.5, SD 5.8, range = 60–82 years), Seidman et al. [62] found that most elderly men with dysthymic disorder had TT levels in the hypogonadal range (i.e., ≤300 ng/dL). Furthermore, their TT levels were lower than in patients with MDD or healthy controls. The authors hypothesized that dysthymic disorder in elderly men may be related to HPG axis hypofunction. This association is believed to be the result of either chronic depression leading to HPG axis blunting, or to HPG axis hypofunctioning leading to low-grade depression [68].

Furthermore, there is some evidence that, compared with controls, T secretion is blunted among older men with severe major depressive disorder (MDD), it appears to normalize after major depressive episode remission [60, 61]. A significant increase in depression during the androgen deprivation treatment period, and a tendency to decline after chemical castration was discontinued has been observed in eugonadal men at risk for prostate cancer who are treated with androgen blockade therapy [69].

No association between depression and T level has been reported in the Massachusetts Male Aging Study, a cross-sectional, population-based multidisciplinary survey of 1,709 normally aging men (aged 39–70 years) [70]. Partially positive results were also reported more recently in the Tromso Study [56]. In this study, lower testosterone levels were associated with subthreshold symptoms of anxiety and depression. The Veterans' Experience Study [71], which investigated a sample of 4,393 veterans who served the U.S. military (mean age 37 years), found small but significant associations between depression and T Level (*r* = 0.04; *P* < 0.01). However, in this latter study, the authors pointed out that the relationship between T level and depression may actually be curvilinear [71].

The causative nature of the relationship between low T levels and depression is uncertain. For example, investigators of the Massachusetts Male Aging Study found a significant interaction between polymorphic CAG repeats sequence encoding a variable-length glutamine chain in the N-terminal transactivation domain of an androgen receptor genetic polymorphism protein, testosterone level, and depression [72]. The CAG repeat length appears to have modulatory effects on androgen action [73, 74], and the associations between depression and testosterone concentration may be mediated by different androgen sensitivity. The psychiatric effects of T may be also mediated through modulation of brain monoamine levels and, in particular, of the serotonergic function [75, 76]. In animal models, T increases cortical serotonin 2A receptor binding densities [77] and, in humans, cortical serotonin 2A receptors decrease with depression and aging [78].

### 3.2. T Treatment of Depression in Older Men

Although the practice of hormone replacement therapy began as long ago as the 18th century, with the use of extractions of reproductive organs of animals to treat a variety of ailments or to enhance the capacity for enjoyment of work and sexual activity [79–81], the role of T therapy for middle-aged and older men with depression is still uncertain (see Tables 3 and 4). T replacement in hypogonadal males generally decreases anger, nervousness, irritability and anxiety [35], and consistently leads to increased sexual interest and activity ([82, 83], see [84] for negative results).

In a randomized, placebo-controlled, double-blind, phase III trial (ClinicalTrials.gov identifier: NCT00696748), 184 men suffering from both the metabolic syndrome and hypogonadism were treated for 30 weeks with either parenteral testosterone undecanoate (TU; 1,000 mg IM TU) or placebo injections [85]. Depression was assessed at the baseline and at 18 and 30 weeks with the BDI. At baseline, depression significantly correlated with the total testosterone level (*r* = −0.16; *P* = 0.03). When comparing the changes over time in patients treated with TU versus the placebo group, there was a significant improvement in depression (mean difference versus placebo after 30 weeks: −2.5 points; 95% CI: −0.9; −4.1; *P* = 0.003). Effects were strongest in men with the lowest baseline total testosterone (<222 ng/dL).

In a sample of 51 hypogonadal men (aged 22 to 60 years) studied for 60 days, T replacement improved positive mood parameters, such as energy, well-being and friendliness, and decreased negative mood parameters including anger, nervousness, and irritability [35]. Direct correlations between serum T and dihydrotestosterone (DHT) with mood scores were only observed in the baseline period, when serum androgen levels were below the normal range. This observation may indicate that it is possible that, once a minimally adequate serum T/DHT level is achieved by T replacement therapy, further increases in serum T/DHT levels do not further contribute to the improvement in mood variables. In a subsequent trial, Wang et al. [82] administered a transdermal T gel formulation to hypogonadal men (227 men aged 19 to 68 years) over a period of 180 days. Mood improved and the improvement was maintained with continued treatment.

Recently, Shores et al. [86] examined the effect of testosterone treatment in older, hypogonadal men (50+ years old) with subthreshold depression in a double-blind randomized controlled trial. Participants received either 7.5 g of testosterone gel or placebo gel daily for 12 weeks, followed by a 12-week open-label extension phase during which all subjects received 7.5 g of testosterone gel. At the end of the double-blind phase, testosterone-treated men had a greater reduction in depression (*P* < 0.05) and a higher remission rate of subthreshold depression (52.9% versus 18.8%, *P* < 0.05) than did placebo-treated men. At the end of the open-label phase, the testosterone group had sustained improvement, while patients who had received placebo in the previous 12 weeks improved, and there were no differences between groups on the number of depressive symptoms reported.

Seidman et al. [87] conducted a six-week double-blind placebo-controlled clinical trial in 23 men with mid-life onset male dysthymic disorder and with low or low-normal testosterone level (TT < 350 ng/dL). After the intervention, the depression decreased significantly more in the testosterone group than in the placebo group (*P* < 0.01).

However, some studies have shown that, in the short and long term [88], T replacement is not superior to placebo in elderly men with low-normal gonadal status, or in men with the lowest BT levels [89–91].

Androgen treatment in eugonadal men has demonstrated subtle changes in sexual arousal, cognition, and mood [36], with a significant increase in manic and aggressive symptoms [92, 93]. However, two studies failed to observe effects of T on mood in healthy men with induced hypogonadism who were given T [94, 95].

To date, little evidence supports the use of androgen therapy in older depressed men [96]. In a study by Perry et al. [97], a subgroup of elderly depressed males (aged 70 and over) improved with T therapy. In a study of 15 elderly eugonadal males with major depressive disorder (MDD, according to the DSM-IV criteria), 5 with early onset MDD, and 10 with late onset MDD, treatment with T cypionate (100 mg/week or 200 mg/week IM for 6 weeks) was efficacious only in some cases of late-onset depression.

Androgen administration in open and blind clinical trials to chronically depressed men or to hypogonadal men with depression refractory to selective serotonin reuptake inhibitors (SSRIs) improved depressive symptoms. Human and animal studies have demonstrated that T treatment may facilitate the antidepressant drug response [98–100]. T augmentation in men with major depression refractory to SSRIs treatment and low or borderline TT levels (200–350 ng/dL) produced significant positive results in short-term treatment (12 weeks) [98, 101], but doubts arose about longer-term treatment (20 weeks) [99].

At the present time, available data do not suggest the use of T in the treatment of depression in PADAM. Data on older men suffering from depression and PADAM are still few and inadequate [102], and the current clinical guidelines for men with low serum T concentration stress that T therapy for depression is irrelevant [12, 103, 104].

Furthermore, T supplementation may be associated with some adverse effects, such as erythrocytosis, acne and oily skin, detection of subclinical prostate cancer, growth of metastatic prostate cancer, and reduced sperm production and fertility [12, 104]. Other, uncommon, adverse events for which there is weak evidence of association with testosterone administration are gynecomastia, male pattern balding (familial), and induction or worsening of obstructive sleep apnea. Formulation-specific adverse effects include fluctuation in mood or libido, pain at injection site, excessive erythrocytosis (especially in older patients), and coughing episodes immediately after the intramuscular injection for intramuscular injections of testosterone enanthate, cypionate, or undecanoate, as well as frequent skin reactions at application site for transdermal patches, and potential risk for testosterone transfer to partner or others in close contact with the individual, and skin irritation for transdermal gel [12].

## 4. Discussion

As Western populations represent an aging society with continuing gains in life expectancy [4], hypogonadism in older men may have significant public health implications [67, 109, 110]. For example, over the last decade, this has led to a significant market growth in T therapies for men 40 years and older [4].

PADAM includes behavioral and depressive symptoms that vary greatly from individual to individual, being the result not only of biological and psychosocial changes, but also of personal ability to adapt to such changes. The efficacy of T therapy in the treatment of depression in elderly hypogonadal men is inconclusive. Research on T replacement therapy for depressive symptoms of PADAM reveals the great variability of the results.

Nonetheless, androgens supplementation may be a useful as adjunctive therapy in depressed hypogonadal men. Several study reported that antidepressants may be associated with sexual dysfunction in adult patients [111], and up to 20% of users may suffer from sexual dysfunction [112]. Sexual dysfunction may also be associated with discontinuation of antidepressants treatment [113]. T treatment may have beneficial effects on sexual functions [83, 89, 94, 103, 114–116]. Recently, Amiaz et al. [83] conducted a 6-week, double-blind, placebo-controlled clinical trial of testosterone gel versus placebo gel in men with MDD who were currently taking a serotonergic antidepressant and exhibited low or low-normal testosterone level. The results indicated that those taking testosterone improved in sexual functions as measured through the International Index of Erectile Function more than those in the placebo arm. Furthermore, the results indicated that the improvement in sexual functioning did not appear to be attributable to improvement in depression.

T treatment may be particularly useful to improve quality of life in elderly hypogonadal men, because its effect on muscular strength [117–119] and may be on cognitive functions [57, 120–122].

However, due to adverse effects associated with T therapy, pretreatment screening for parameters related to potential risks of testosterone supplementation is essential. T supplementation is contraindicated in individuals with hematocrit of 52% and over [123, 124], prostatic carcinoma, an androgen sensitive-tumor, and in cases of mammary carcinoma in men [12, 103].

In conclusion, despite the causative nature of the relationship between low T levels and depression is uncertain, many hypogonadal men suffer from depression and vice versa. Supplementation with testosterone failed to show sound evidence of effectiveness in the treatment of depression. Nevertheless, T supplementation has proved to be effective on some domains significant for the quality of life of patients with PADAM. Those effects may partially mediate the effects on depressive symptomatology reported in some trials. Thus, the overall improvement in well-being and health, related to the quality of life in aging males with partial androgen deficiency, may have a positive impact on their mood.

## Figures and Tables

**Table 1:** Naturalistic and cross-sectional studies in nonselected samples of patients.

Author, year	Type of study	Sample size	Age	Outcome measures	Results
Barrett-Connor et al. [64]	Cross-sectional population-based study	856 men	Range: 50–89 yrs	Association between BDI and BT or DHT	BDI scores were inversely associated with BT and DHT. BT were 17% lower for men with categorically defined depression than levels observed in all other men.

T'Sjoen et al. [105]	Naturalistic	236 male outpatients in 1997 and 192 in 2000	All patients were 70+ yrs. Range: 73.5–78.5 yrs in 1997; 76.0–81.0 yrs in 2000	Association between GDS and TT, FT, estradiol, DHEAS, AR gene CAG-repeat length	No relationship between the GDS and FT or TT in 1997. The GDS did not correlate with AR gene CAG-repeat length. The GDS of 1997 correlated significantly with (free) estradiol and with DHEAS. The analysis in 2000 confirmed a lack of association between GDS and androgen levels or AR gene CAG-repeat length. The association between GDS and (free) estradiol and DHEAS was not observed in the subgroup of men studied in 2000. Changes in FT and serum (free) estradiol levels between 1996 and 2000 were not related to GDS score in 2000.

Strasser et al. [55]	Naturalistic	48 patients with cancer who had received no major antineoplastic intervention for at least 2 weeks	Range: 20–79 yrs; Median: 59 yrs	Association between HAM-D and hypogonadism	64% of patients had hypogonadism, which was correlated with HAM-D score.

Spetz et al. [43]	Cross sectional	370 men	Range: 55 to 75 yrs; median: 62 yrs	Blood concentrations of T and BT	Men reporting deterioration in work performance had significantly lower T (16.6 nmol/L) and BT (6.9 nmol/L) than men without this problem (18.8 nmol/L and 7.9 nmol/L, resp.). Men reporting decreased strength and/or endurance had lower concentrations of BT than men without the same complaint (7.2 nmol/L and 8.0 nmol/L, resp.). Men suffering from hot flushes had lower T (15.1 nmol/L) and BT (6.58 nmol/L) compared with men who either had flushes but were not bothered by them or men without any flushes (17.6 nmol/L and 7.63 nmol/L).

Kratzik et al. [54]	Naturalistic	669 manual workers	Range: 43–67 years	Association between BT, BMI and BDI scores	There was a U-shaped (quadratic) association between BT and BDI. Obese and underweight men with high BT had an increase BDI score. Men with low BT levels had an increased BDI score and eugonadal men with normal T levels have the lowest risk of depression.

Morsink et al. [53]	Prospective cohort udy	2855 well-functioning elderly men (1406) and women (1449)	All aged aged 70–79 yrs; Mean: 73.8 yrs (men); 73.5 yrs (women)	Associations between TT, FT, DHEAS and CES-D scores	Significant inverse association between DHEAS and the CES-D for men and women. In men, there was a borderline significant inverse association between TT and depressive symptoms. For men, those in the lowest quartile of DHEAS and TT had significantly more depressive symptoms than those in the other quartiles.

Makhlouf et al. [52]	Naturalistic	157 men referred to an erectile dysfunction specialty clinic	Range: 21–85 yrs; Median = 53 yrs	Scores of 22 or higher on the CES-D (overt depression)	Hypogonadal men were 1.94 times more likely to have overt depression scores compared to eugonadal counterparts.

Fukai et al. [50]	Cross-sectional	108 men and 100 women	Range: 70–95 yrs; Mean: 82 ± 7 yrs (men); 81 ± 6 yrs (women)	Associations between GDS and TT, FT, DHEA, DHEAS, Estradiol	Linear regression model of hormone levels on functional scores unadjusted and adjusted for age, and age and body mass index indicated that in men the GDS was not associated with hormone levels.

Hintikka et al. [47]	Naturalistic	59 men with adverse mental symptoms (AMS) and 57 asymptomatic (AS) men from the Finnish National Population Register	Mean: 55.9 ± 8.6 yrs for AMS and 56.3 ± 10.4 yrs for AS	Hypogonadism (FT < 160 pmol/L)	AMS more often had hypogonadism; BDI score and HAM-D correlated negatively with FT in the entire sample.

Wu et al. [7]	Naturalistic	Random population sample of 3,369 men at eight European centers	Mean: 59.7 ± 0.3 yrs	Associations between TT and FT, and items from the SF-36 and the BDI	Sadness (SF-36), loss of energy (BDI), and fatigue were significantly associated with FT but not with TT. The probability of symptoms increased with decreased levels of T. The thresholds for TT were approximately 160 pmol/L for both sadness and fatigue.

Jankowska et al. [49]	Cross-sectional	Study population: 163 men with stable systolic chronic heart failure (CHF), and 316 healthy men living in the same area	Range: 35–80 yrs (controls) Mean: 60 ± 10 yrs (CHF)	Associations between BDI and TT or DHEAS	There were no differences in serum TT levels between the healthy men and the men with CHF when evaluated according to BDI score. Men with CHF had lower serum DHEAS as compared with healthy men in subsequent groups according to BDI score. Lower TT and DHEAS were associated with depressive symptoms in men with CHF.

Wong et al. [106]	Naturalistic	1147 community-dwelling elderly men, aged	65+ yrs	Associations between GDS, DHEA, TT, FT, estradiol, and SHBG	DHEA was significantly associated with GDS score. No association was seen between depressive symptoms and TT, FT, estradiol levels or SHBG levels.

Berglund et al. [56]	Naturalistic	3413 men participating in the fifth Tromsø study in 2001	Range: 60 ± 14 yrs	Associations between the Hopkins Symptom Checklist-10 (SCL-10) scores and T	Men with T levels in the lowest 10th percentile, had increased SCL-10 score compared to men with higher T levels. However, men with more pronounced symptoms indicating mental disorder did not have lower testosterone levels.

Halabi et al. [63]	Cross-sectional	104 men with COPD; 36 of whom had significant depressive symptoms (GDS ≥ 11)	All 55+ yrs. Mean: 66 ± 1 (depressed), 71 ± 1 (nondepressed):	Associations between GDS and hypogonadism	Prevalence of hypogonadism was greater in patients with severe depressive symptoms (GDS ≥ 19) than in those with mild depressive symptoms (GDS = 11–18) (62% versus 26%). After controlling for confounders, however, gonadal state was not associated with severe depressive symptoms.

T: testosterone; BT: bioavailable testosterone; FT: free testosterone; DHEA: dehydroepiandrosterone; DHEAS: dehydroepiandrosterone sulphate; SHBG: sex hormone-binding globulin; BMI: body mass index; HAM-D: Hamilton scale for depression; BDI: Beck depression inventory; CES-D: center for epidemiologic studies depression scale; GDS: geriatric depression scale.

**Table 2:** Naturalistic and cross-sectional studies in patients with depressive disorders.

Author, year	Type of study	Sample size	Age	Outcome measures	Results
Steiger et al. [61]	Pre-post analyses	12 male patients with MDD	Mean: 46.4 ± 11.3 yrs	Change in nocturnal secretion of T	Nocturnal secretion of T increased after remission of depression.

Booth et al. [71]	Naturalistic	4,393 men from the Vietnam Experience Study	Range: 30–48 yrs; Mean = 37 yrs	Associations between T and depression measured with the items from the Diagnostic Interview Schedule	T-squared has a significant relationship with depression, indicating a curvilinear relationship. Among men whose testosterone is below 590 ng/dL, each increase in the testosterone level is associated with less depression. Among men whose testosterone is above this value, each increase in the hormone is associated with greater depression.

Schweiger et al. [60]	Cross-sectional	15 male inpatients with moderate to severe MDD and 22 healthy comparisons	Range: 22–85 yrs; 48 ± 15 for MDD versus 53 ± 18 for comparisons	Differences in T between groups	Daytime T, nighttime T, and 24-hour mean T secretion were significantly lower in the MDD inpatients.

Seidman et al. [62]	Cross-sectional	32 elderly men with dysthymic disorder, 13 elderly men with MDD, and 175 nondepressed comparisons	Range: 60–82 yrs	Differences in TT	TT was lower in elderly men with dysthymic disorder than in men with MDD and men without depressive symptoms.

Shores et al. [67]	Naturalistic	278 men with consistently normal or low T levels (TT ≤ 200 ng/dL; or FT ≤ 0.9 ng/dL) at baseline and during a 2-year follow-up period	45+ yrs	Differences in prevalence of ICD-9-CM depressive illness	The hypogonadal men had an increased occurrence of diagnosed depressive illness (21.7% versus 7.1%).

McIntyre et al. [44]	Cross-sectional	44 depressed and 50 nondepressed men	Range: 40–65 yrs; depressed= 52.0 ± 7.1; controls= 50.8 ± 6.5	Differences in T levels	Depressed men had lower BT levels and TT levels than controls. Biochemical hypogonadism (i.e., BT level ≤ 2.4 nmol/L or TT level ≤ 12.14 nmol/L) was also more prevalent in depressed men than in non-depressed controls (34% versus 6% and 61% versus 14%, resp.).

Ponholzer et al. [51]	Naturalistic	247 men of a population-based study	Mean: 75.7 yrs	Differences in T levels and DHEAS	T levels were not associated to prevalence or incidence of depression or dementia.

T: testosterone; BT: bioavailable testosterone; FT: free testosterone; DHEA: dehydroepiandrosterone; DHEAS: dehydroepiandrosterone sulphate; SHBG: Sex hormone-binding globulin; BMI: body mass index; HAM-D: Hamilton scale for depression; BDI: Beck depression inventory; CES-D: center for epidemiologic studies depression scale; GDS: geriatric depression scale; MDD: major depressive disorder.

**Table 3:** Intervention studies in samples of patients with depressive symptoms.

Author, year	Type of study	Sample size	Age	Treatment	Outcome measures	Results
Wang et al. [35]	Pre-/postanalyses	51 hypogonadal men	22–60 yrs old	18 received T enanthate 200 mg im every 20 days, 16 received sublingual T cyclodextrin (SLT) at a dose of 2.5 mg three times daily, and 17 received SLT at a dose of 5.0 mg three times daily. The total treatment period was 60 days	Change in scores in single items on a 7-point Likert rating scale measuring anger, alertness, irritability, energy, sadness, tiredness, friendliness, nervousness, and well-being	T replacement led to significant decreases in anger, irritability, sadness, tiredness, and nervousness, and significant improvement in energy, friendliness, and well-being in all subjects as a group. Baseline serum T was positively correlated with friendliness and well-being, and negatively correlated with nervousness, irritability and tiredness. After T replacement these correlations disappeared.

Wang et al. [35]	Pre-/postanalyses	30 hypogonadal men	All 18+ years old	Sublingual T cyclodextrin 5 mg 3 times daily for 6 months	—	The patients were less nervous and more alert, friendly, and energetic during the 6-month treatment period compared with baseline.

Alexander et al. [107]	Cross-sectional	33 hypogonadal men receiving T replacement. 10 eugonadal men receiving T and 19 eugonadal men not administered T	Range: 19–60 yrs Mean: 41.1 yrs (hypogonadal); 33.4 yrs (eugonadal men receiving T); 32. 7 (eugonadal men not administered T)	Eugonadal men received weekly i.m. injections of testosterone enanthate (TE) (200 mg). Hypogonadal men were treated either with 200 mg TE every 20 days or with 2.5 or 5.0 mg sublingual testosterone cyclodextrin 3 times daily	Change in POMS scores after 6 weeks of treatment.	T had positive effects on mood in hypogonadal men, but did not have any effects on mood in eugonadal men.

Wang et al. [82]	Double-blind RCT	227 hypogonadal men	About 3.9–11.0% of the subjects were <35 yrs, 23.3–36.8% were between 35–49 yrs, 55.1–57.5% were between 50–64 yrs, and 3.9–8.2% were 65+ yrs in the 3 initial treatment groups	In the first 3 months the subjects were randomized to receive 50 mg/day T gel in 5 g gel, 100 mg/day T gel in 10 g gel, or 2 nonscrotal patches delivering 5 mg/day (T patch). In the following 3 months, the subjects were administered 1 of the following treatments: 50 mg/day T gel, 100 g/day T gel, 5.0 mg/day T patch, or 75 mg/day T gel in 7.5 g gel	Sexual function and mood were assessed before clinic visits on day 0 and on days 30, 60, 90, 120, 150, and 180 during gel and patch application	All subjects as a group showed improvement in positive mood. Similarly, the negative mood summary scores showed significant decreases without showing between-group differences.

Pope et al. [93]	RCT	56 men	Range: 20–50 yrs	Testosterone cypionate for 6 weeks in doses rising to 600 mg/wk and placebo for 6 weeks, separated by 6 weeks of no treatment	Differences in YMRS and HAM-D scores	84% of those who received 600 mg/wk of testosterone cypionate exhibited minimal psychiatric effects (YMRS ≤ 10), 12% became mildly hypomanic (YMRS= 10–19), and 4% became markedly hypomanic (YMRS ≥ 20). The HAM-D remained low, with no changes during T administration or withdrawal.

O'Connor et al. [108]	RCT	30 healthy men and 8 hypogonadal male patients	Healthy males: Range: 19–45 yrs; Mean: 28.2 yrs Hypogonadal men: Range: 23–40 yrs; Mean: 30.8 yrs	15 eugonadal-men received 200 mg i.m. T enanthate once weekly for 8 weeks, 15 received 200 mg i.m. 0.9% sodium chloride solution weekly for 8 weeks. The hypogonadal group received 200 mg i.m. T enanthate biweekly for 8 weeks	Differences in depression-dejection dimension of the POMS	Significant main effects were found for time, group, and for time x group interaction. Multiple comparisons found that the significant group effect was accounted for by significantly higher levels of total mood disturbance in the hypogonadal group than the eugonadal-treated and eugonadal-placebo groups. However, there was a significant reduction in total mood scores in the hypogonadal group by weeks 1-2 explaining the significant interaction effect.

Almeida et al. [69]	Pre-/postanalyses	40 men with prostate cancer treated with androgen blockade therapy	Range: 44–83 yrs; Mean = 72.4 yrs	Androgen blockade therapy (flutamide and leuprolide) for 36 weeks and subsequently followed up for another 18 weeks after discontinuation	Change in BDI scores	BDI scores increased significantly during the active treatment and declined somewhat thereafter. However, the number of people with clinically significant depressive symptoms did not change significantly.

Schmidt et al. [95]	Double-blind RCT	31 healthy adult men with no history of psychiatric illness or substance or anabolic steroid abuse	Range: 18–45 yrs; Mean: 30.8 ± 5.8 yrs	Leuprolide acetate (Lupron) 7.5 mg im every 4 weeks for 3 months. After the first month of Lupron alone, all men received (in addition to Lupron) testosterone enanthate (200 mg i.m.) or placebo every 2 weeks for 1 month each in a crossover design.	Changes in BDI scores	BDI scores significantly increased during Lupron plus placebo compared with baseline and Lupron plus testosterone.

Kenny et al. [102]	RCT	11 men with early cognitive decline and bioavailable T levels below 128 ng/dL	Range: 73–87 yrs; Mean: 80 ± 5 yrs	intramuscular testosterone (200 mg every 3 weeks) or placebo for 12 weeks	Changes in GDS scores	No significant changes were found in depression following T supplementation

Haren et al. [90]	Double-blind RCT	76 healthy men with at least two symptoms on the ADAM, a FT index (FTI) of 0.3–0.5 and TT greater than 8 nmol/L	Range: 60–86 yrs Mean: 68.5 ± 6 yrs	80 mg twice daily of testosterone undecanoate—TU (39 subjects) or identical placebo (37 placebo) for 12 months	Differences in GDS scores	From baseline to month-6 there was a significant effects of treatment on depression. No clinically relevant differences on the GDS between the testosterone and placebo group.

Gray et al. [94]	RCT	60 healthy men	Range: 60–75 yrs	Monthly injections of long-acting GnRH agonist to suppress endogenous T production and randomization to one of five doses (25, 50, 125, 300, and 600 mg) of testosterone enanthate weekly for 20 weeks	Changes in HAM-D and YMRS	Baseline depression and mania were not correlated with log FT levels. Changes in mood did not differ by group and were not significantly correlated with FT or TT.

Giltay et al. [85]	Double-blind RCT	184 men with TT below 12.0 nmol/L or FT below 225 pmol/L, and a diagnosis of the MetS	Range: 35–69 yrs; Mean: 52.1 ± 9.6 yrs	30 weeks with either parenteral testosterone undecanoate (1,000 mg i.m., at baseline, and after 6 and 18 weeks) or placebo injections	Association between BDI and TT. Changes in BDI scores	At baseline, BDI scores significantly correlated with TT (*r* = −0.16). More improvements in BDI for those treated with T (mean difference versus placebo after 30 weeks: −2.5 points).

Aloisi et al. [88]	Open label	9 opioid-induced hypogonadic men. T less than 2-3 ng/mL in at least two determinations in the previous 3-4 months	Range: 38–74 yrs; Mean: 59.0 ± 4.4 yrs	One-month supply of testosterone gel, a hydroalcoholic compound containing 50 mg testosterone in 5 g gel in each sachet for 1 year	Change in CES-D scores from baseline	CES-D showed no significant change from baseline to follow-up assessments at 3, 6 and 12 months.

T: testosterone; BT: bioavailable testosterone; FT: free testosterone; DHEA: dehydroepiandrosterone; DHEAS: dehydroepiandrosterone sulphate; SHBG: Sex hormone-binding globulin; BMI: body mass index; HAM-D: Hamilton scale for depression; BDI: Beck depression inventory; CES-D: center for epidemiologic studies depression scale; GDS: geriatric depression scale; POMS: profile of mood states; YMRS: young mania rating scale; MDD: major depressive disorder; MetS: metabolic syndrome.

**Table 4:** Intervention studies in samples of patients with depressive disorders.

Author, year	Type of study	Sample size	Age	Treatment	Outcome measures	Results
Seidman and Rabkin [98]	Open-label	5 depressed men who had low T levels and had not responded to an adequate SSRI trial	Range: 34–50 yrs; Mean: 40 ± 5.9 yrs	400 mg testosterone replacement biweekly for 8 weeks	Changes in HAM-D scores from baseline	Significant recovery from major depression following T augmentation.

Perry et al. [97]	RCT	15 elderly eugonadal males with MDD (HAM-D > 18)	Mean: 61.3 ± 7.6 yrs	Following a single-blind 2-week placebo lead-in, patients were randomly assigned to treatment with either a physiologic dose of testosterone cypionate (TC), 100 mg/week, or supraphysiologic dose of 200 mg/week i.m. for 6 weeks	Changes in HAM-D scores	42% decrease in the mean HAM-D scores. However, the majority of the change was due to improvement in the late-onset depression patients. The TC dose did not affect the response.

Pope et al. [101]	Double-blind RCT	22 MDD patients with morning serum TT of 350 ng/dL or less who were receiving antidepressant treatment	Range: 30–65 yrs; Mean: 48.9 ± 8.5 (T group) and 49.5 ± 9.8 (placebo group)	12 men received 1% testosterone gel (10 g/day) and 10 received placebo	BDI, HAM-D, CGI-S	Subjects receiving testosterone gel had significantly greater improvement on the HAM-D than subjects receiving placebo. These changes were noted on both the vegetative and affective subscales of the HAM-D. A significant difference was also found on the CGI-S but not on the BDI.

Orengo et al. [99]	RCT	12 hypogonadal men who were receiving antidepressants (on appropriate dose) for a minimum of 6 weeks	Range: 52–80 yrs; Mean: 63 ± 8.5 yrs	Placebo or active T gel 1% at a dose of 5 g for 24 weeks	Differences in HAM-D scores	There was a significant improvement in HAM-D at 12 weeks of testosterone treat- ment as compared to baseline. However, there was no statistical difference between placebo and testosterone treatments.

Seidman and Roose [84]	Double blind RCT	30 men with low and low-normal T levels (i.e., total T <or = 350 ng/dL) and MDD	Range: 33–71 yrs; Mean: 52 ± 8 yrs	Weekly intramuscular injections of either T enanthate 200 mg or placebo for 6 weeks	Differences in HAM-D scores	The HAM-D scores decreased significantly in both T and placebo groups, and there were no significant between-group differences.

Shores et al. [86]	Double-blind RCT followed by an open-label extension phase	33 men with TT levels of ≤280 ng/dL and subthreshold depression (dysthymia or minor depression, according to DSM-IV)	All 50+ yrs old; Mean: 57.1 ± 5.7 yrs (T); 61.7 ± 7.0 yrs (placebo)	Either 7.5 g of testosterone gel (17 men) or placebo gel (16 men) daily for 12 weeks, followed by a 12-week open-label extension phase during which all subjects received 7.5 g of testosterone gel	Differences in HAM-D scores	At the end of the double-blind phase, testosterone-treated men had a greater reduction in HAM-D scores and a higher remission rate of subthreshold depression (52.9% versus 18.8%) than did placebo-treated men. At the end of the extension phase, there were no significant between-group differences in the remission rate of depression between the original testosterone group and the original placebo group (58.8% versus 68.8%, resp.). .

Seidman et al. [87]	Double-blind RCT	23 men with dysthymic disorder and with low or low-normal T level (i.e, TT < 350 ng/dL)	Mean: 50.6 ± 7.0 yrs	200 mg of testosterone cypionate im or placebo every 10 days for 6 weeks	Difference in HAM-D and CGI-I	HAM-D score decreased significantly more in the T group (7.46 ± 4.56) than in the placebo group (1.8 ± 4.13). Patients in the T group were more likely to remit (53.8% versus 10%) than patients in the placebo group.

Pope et al. [91]	Double-blind RCT	100 medically healthy adult men with MDD showing partial response or no response to an adequate SSRI trial during the current episode and a screening TT ≤ 350 ng/dL	Mean: 50.6 ± 8.2 and 49.9 ± 7.1, respectively for those treated wih T and those in the placebo group	Placebo gel (50 men) or testosterone gel (50 men) at 5 g/day. If the testosterone level at week 1 exceeded the physiologic range (91070 ng/dL), the investigator reduced the dose of gel to 2.5 g/day; if the level was 500 ng/dL or lower, then the investigator issued instructions to raise the dose to 10 g/day	Difference in HAM-D and MADRS	No significant difference in the antidepressant effects of T and placebo gel augmentation

T: testosterone; BT: bioavailable testosterone; FT: free testosterone; DHEA: dehydroepiandrosterone; DHEAS: dehydroepiandrosterone sulphate; SHBG: Sex hormone-binding globulin; BMI: body mass index; HAM-D: Hamilton scale for depression; BDI: Beck depression inventory; CES-D: center for epidemiologic studies depression scale; GDS: geriatric depression scale; POMS: profile of mood states; YMRS: young mania rating scale; CGI-S: clinical global impression scale-severity; CGI-I: clinical global impression scale-improvement; MDD: major depressive disorder; MetS: metabolic syndrome.
